# *Suaeda salsa* Root-Associated Microorganisms Could Effectively Improve Maize Growth and Resistance under Salt Stress

**DOI:** 10.1128/spectrum.01349-22

**Published:** 2022-08-11

**Authors:** Yongdong Wang, Qinghua Sun, Jiai Liu, Lingshuai Wang, Xiaoliang Wu, Zhenyi Zhao, Ningxin Wang, Zheng Gao

**Affiliations:** a State Key Laboratory of Crop Biology, Shandong Agricultural University, Tai'an, China; b College of Life Sciences, Shandong Agricultural University, Tai'an, China; c College of Plant Protection, Nanjing Agricultural University, Nanjing, China; d Taishan University, Tai'an, China; e College of Plant Protection, Shandong Agricultural University, Tai'an, China; Beijing Forestry University

**Keywords:** maize, microbial communities, physiological responses, salt-tolerant microorganisms

## Abstract

Root-associated microorganisms are widely recognized as playing an important role in mitigating stress-induced damage to plants, but the responses of rhizosphere microbial communities after inoculation and their relationship with plant responses remain unclear. In this study, the bacterium Providencia vermicola BR68 and the fungus *Sarocladium kiliense* FS18 were selected from among 91 strains isolated from the halophyte *Suaeda salsa* to interact with maize seedlings under salt stress. The results showed that compared with NaCl-only treatment, inoculation with strains BR68 and FS18 significantly improved the growth, net photosynthetic rate, and antioxidant enzyme activities of maize; significantly reduced proline content and generation rate of reactive oxygen species (ROS); and alleviated oxidative stress and osmotic stress. Moreover, inoculation with these two strains increased the activities of soil microbiome enzymes such as sucrase, catalase, and fluorescein diacetate hydrolase, which improved maize physiologies and promoted maize growth under salt stress. In addition, these inoculated strains significantly affected the abundance of certain genera, and the correlation trends for these genera with soil properties and maize physiologies were similar to those of these inoculated strains. Strain BR68 was indirectly associated with bacterial communities through BR-specific biomarkers, and bacterial communities and soil properties explained most of the variation in maize physiologies and growth. Inoculation of strain FS18 was directly associated with variations in soil properties and maize physiologies. The two strains improved maize growth under salt stress and alleviated stress damage in maize in different ways. The links among salt-tolerant microorganisms, soil, and plants established in this study can inform strategies for improving crop cultivation in salinized lands.

**IMPORTANCE** This study demonstrates that halophyte root-associated microorganisms can promote crop tolerance to salt stress and clarify the mechanism by which the strains work in rhizosphere soil. The links among salt-tolerant microorganisms, soil, and plants established in this study can inform strategies for improving crop cultivation in salinized lands.

## INTRODUCTION

Worldwide, one of the most serious problems associated with soil is salinization, which has a substantial impact on crop growth and development. It has been estimated that more than 800 million hectares arable in the world are affected by soil salinization and that the area of salinized land will continue to rise in the future (1, 2). Salt stress can reduce crop yields by up to 50% or more, resulting in negative ecological and socioeconomic consequences (3). Although salinized soils can be partially remediated by certain physical or chemical means, the high cost of remediation prohibits its widespread application (4). As a result, improving the salt tolerance of crops and increasing the yield of crops under salt stress has become an important issue to address for the sustainable and efficient development of agriculture worldwide.

High concentrations of salt in soils can cause osmotic stress due to the inability of plant roots to absorb water normally, and cause an excessive uptake of sodium ions that poison cells and cause nutrient imbalances (5, 6). Osmotic stress and ionic stress lead to the accumulation of a large amount of reactive oxygen species (ROS) in plants, resulting in damage to membrane structures and disruption of intracellular material balances, and inducing biochemical metabolic disorders (7, 8). To alleviate the damage caused by salt stress to plants, some researchers have tried to improve the genetics of plants to improve their salt tolerance. Mutations in certain genes have been reported to increase the resistance of plants to salt stress (9, 10). However, at present, the cultivation of new varieties that show favorable salt resistance properties requires a relatively long period of time, which introduces some inconveniences. In addition, green additives such as biochar and microorganisms have been proven to be effective in alleviating the stress caused by salinity to plants, which has attracted an increasing amount of attention from researchers (11–13).

The plant microbiome, known as the second genome of plants, not only contributes to plant growth and development, but also participates in plant immunity (14–16). It is currently a popular research topic. Different plants can recruit different microorganisms, and the complex and dynamic interactions between microorganisms and plants under abiotic stress can promote crop growth and alleviate stress damage without harming the environment. Previous studies have shown that endophytic or rhizosphere microorganisms of some halophytes improve the salt tolerance of plants. Plant growth-promoting rhizobacteria (PGPR) isolated from salt-stressed tomato rhizospheres have been shown to promote tomato growth under salt stress by reducing the Na^+^ content in tomato plants and increasing the enzyme activities of tomato plants and soil (3). Krishnamoorthy et al. (17) isolated arbuscular mycorrhizal fungi (AMF) from the rhizosphere of *Phragmites* sp., which could synergize with associated bacteria to promote plant growth, root colonization, and nutrient accumulation under salt stress. Although some studies have shown that the rhizosphere microorganisms of halophytes can improve the salt tolerance of other heterologous crops, their mechanism of action is still unclear.

Maize is an important crop that is widely used to provide food for humans and animals. As the world’s population grows, so does its demand for maize. However, the yield of this indispensable crop is strongly impacted by natural and man-made factors, and soil salinization is one of the crucial factors affecting the growth and yield of maize (18, 19). *Suaeda salsa* is an important halophyte that grows widely in coastal saline-alkaline lands. Our previous studies have shown that in the saline-alkaline lands of the Yellow River Delta, the soils planted with crops such as wheat and rice have completely different microbial communities from the nearby soils planted with *Suaeda salsa* (20). Therefore, we hypothesized that microorganisms obtained from the rhizosphere of the halophyte *Suaeda salsa* could also potentially enhance the salt resistance of maize. Based on this, maize was used as the host plant, and salt-tolerant bacteria isolated from *Suaeda salsa* were used as inoculated strains in this study. The purpose of the research was to (i) explore the effect of inoculation of strains from the *Suaeda salsa* rhizosphere on maize growth and salt tolerance; (ii) explore the effect of these inoculated strains on soil properties and maize rhizosphere microbial communities; and (iii) explore the relationships among these inoculated strains, soil properties, soil microbial communities, and maize physiologies and their contributions to changes in maize growth and salt tolerance.

## RESULTS

### Isolation and filtration of salt-tolerant microorganisms.

Microorganisms were isolated from the root and rhizosphere soil of *Suaeda salsa* in an LB medium containing 850 mM NaCl. A total of 91 strains were screened, of which 28 were from the root and 63 were from the rhizosphere soil. On the premise that the isolated strains could grow in a low-salt environment (85 mM NaCl), we further screened 33 strains to conduct pot pre-experiment. We determined root length and plant height of maize seedlings 21 days after inoculation with the strains. The results showed that maize growth inoculated with strain FS18 increased most significantly, with plant height and root length reaching 25.11 cm and 14.52 cm, respectively, followed by BR68, which were 24.15 cm and 13.52 cm, respectively (Table S1). Therefore, these two strains were selected for subsequent experiments.

### Identification and properties of BR68 and FS18.

**FIG 1:**
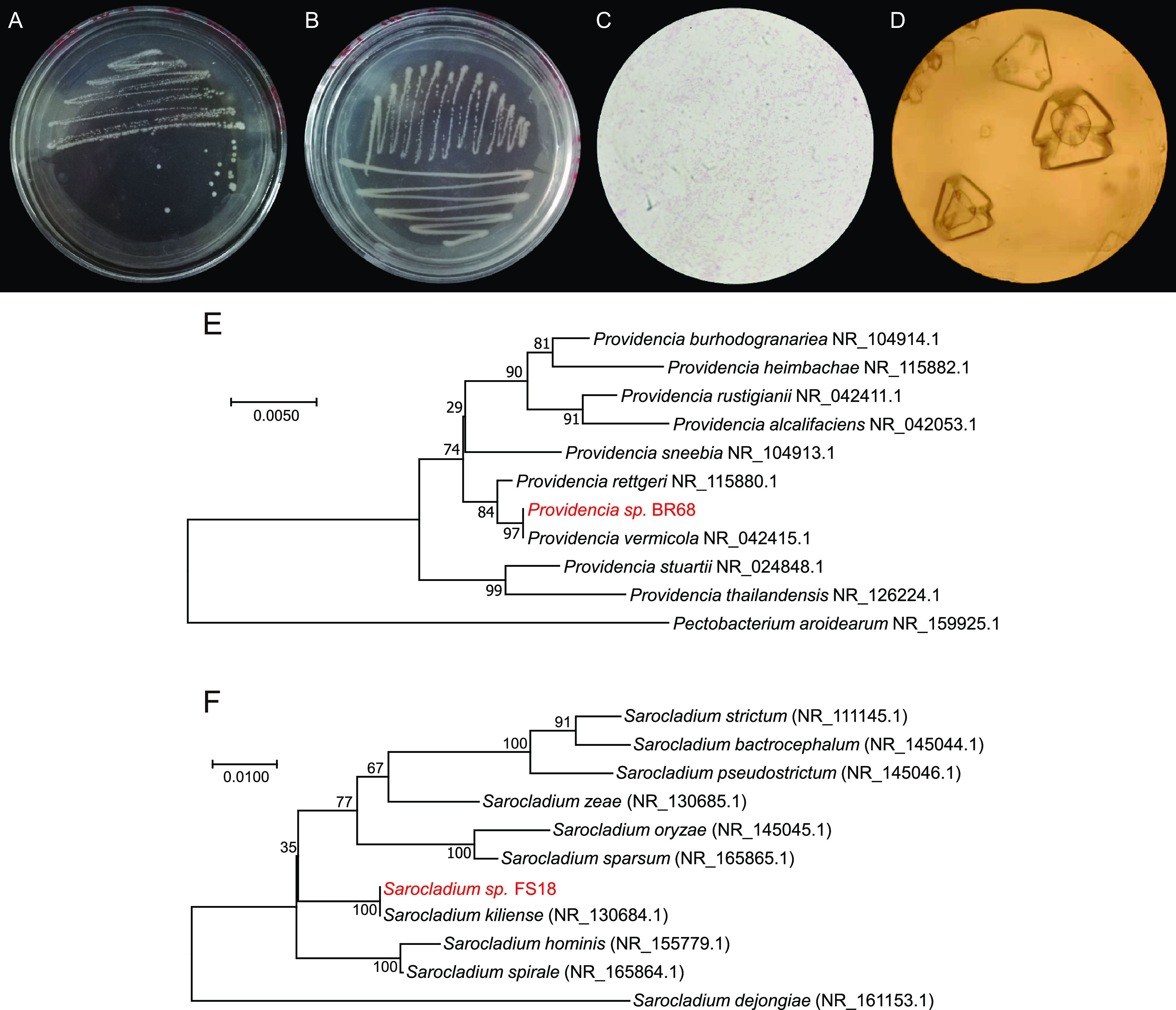
Colony morphology of (A) strain BR68 and (B) strain FS18 on LB medium containing 200 mM NaCl. Morphology of (C) strain BR68 and (D) strain FS18 under microscope. A phylogenetic relationship of strain (E) BR68 and (F) strain FS18 identified on the basis of 16S rDNA gene sequences. The branching pattern was generated by the neighbor-joining method. One thousand times bootstrap analysis for evaluation of phylogenetic tree topology was also calculated. Numbers indicate 0.005 Knuc units.

Strain BR68 came from the root and could grow in a 1,700-mM NaCl environment, while strain FS18 came from the rhizosphere soil and could grow in a 3,400-mM NaCl environment. Strains BR68 and FS18 grew best when the NaCl concentration was 850 mM, with OD_600_ reaching 0.704 and 1.150, respectively. In the above-mentioned LB medium, the colonies of BR68 were white, round, and smooth, and the colonies of FS18 were milky white, irregular, and dry (Fig. 1A and B). BR68 was found to be a Gram-negative bacterium with a short rod shape. FS18 produced mycelium and round spores (Fig. 1C and D). By combining the physicochemical characteristics of the strain with the phylogenetic analysis of its 16S rDNA sequence, BR68 was identified as Providencia vermicola and FS18 was identified by its internal transcribed spacer (ITS) sequence as *Sarocladium kiliense* (Fig. 1E and F, Table S2).

### Effects of inoculated strains on maize plants and soil under salt stress.

**FIG 2:**
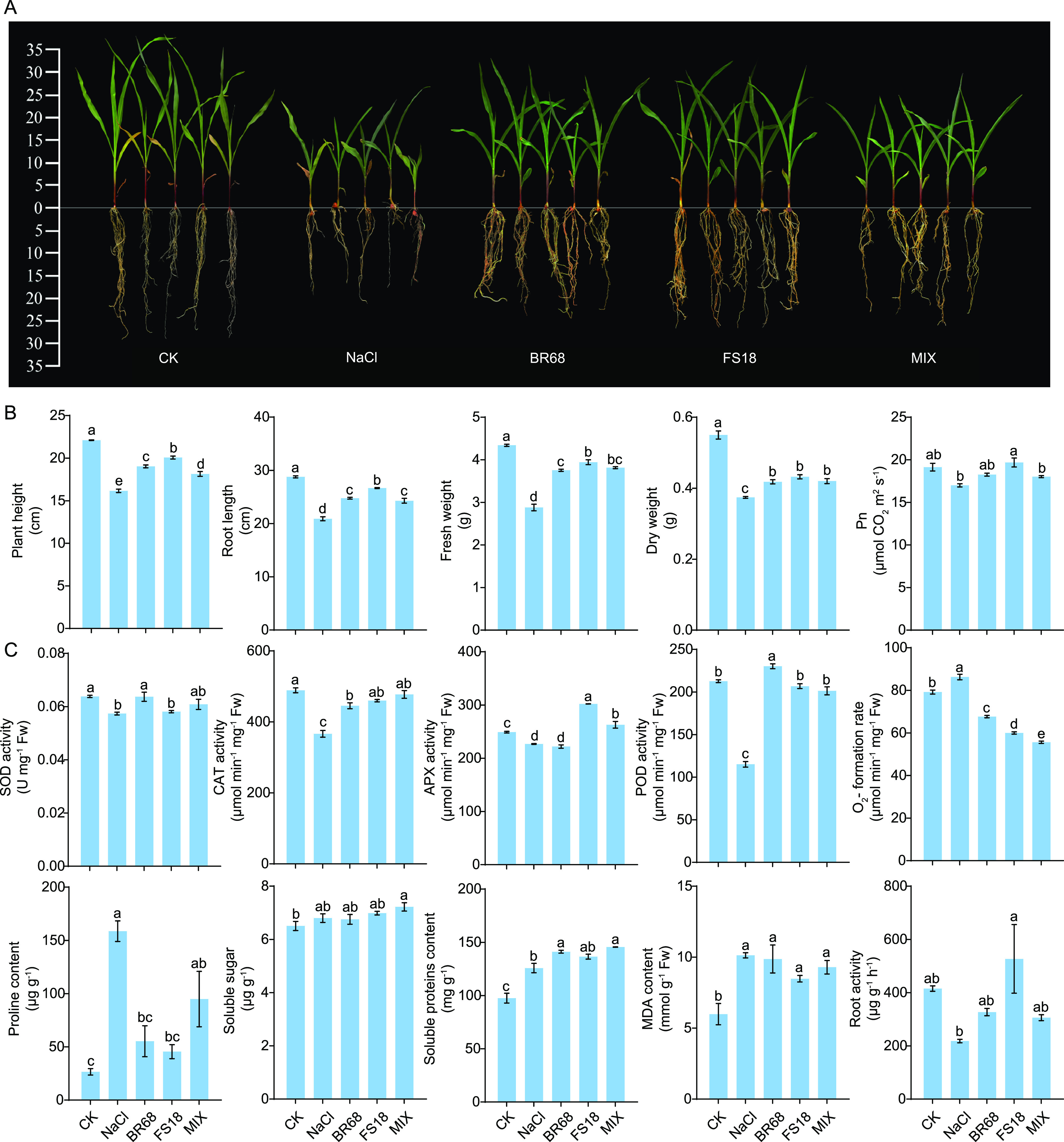
(A) Photos of maize seedlings, (B) plant growth, and (C) physiologies under different treatments after 21 days of application. Average ± standard error from three separate replicates. Values with different letters are significantly different at *P* ≤ 0.05 by variance analysis.

To study the effect of the screened strains on maize growth under salt stress, we carried out a maize pot experiment, including no-salt stress treatments (CK), salt-only stress treatment (NaCl), and treatments with single or mixed applications of these inoculated strains under salt stress (BR68, FS18, MIX). Since these two strains barely grow in the absence of NaCl, we did not set up a treatment to inoculate the two strains under normal conditions. After 21 days of treatment application, the addition of NaCl (NaCl treatment) resulted in a significant inhibition of maize growth, including inhibition of plant height, root length, fresh weight, and dry weight (*P < *0.05), compared with the control. The net photosynthetic rate of leaves also significantly decreased (*P < *0.05). However, the inhibition of maize growth was significantly alleviated by the single and mixed applications of strains BR68 and FS18 (Fig. 2A and B). Compared with the NaCl treatment, the net photosynthetic rate of maize increased by 6.9%, 13.6%, and 6.3% in the BR, FS, and MIX treatments, respectively. The dry weight also increased by 11.8%, 15.5%, and 12.3%, respectively. In addition, the photograph of a maize plant after inoculation with the two strains, showed that the root system was significantly more developed (Fig. 2A).

To further evaluate the effect of these inoculated strains on maize plants under salt stress, we determined a range of maize physiological parameters such as antioxidant enzyme activities. The results showed that the activities of superoxide dismutase (SOD), catalase (CAT), ascorbate peroxidase (APX), and peroxidase (POD) were significantly reduced (*P < *0.05) and ROS was significantly increased (*P < *0.05) in maize leaves in the NaCl treatment compared with the control. However, the hydrogen peroxide scavenging enzyme (CAT and POD) activities of maize in the BR, FS, and MIX treatments were significantly increased (*P < *0.05) compared with the NaCl treatment and almost recovered to the control level (Fig. 2C). In addition, SOD activity was increased by 11% in the BR treatment compared with the NaCl treatment, and APX activity was increased by 33.2% in the FS treatment. ROS were significantly lower (*P < *0.05) in all three treatments compared with the NaCl treatment, even below the control level (Fig. 2C). For the osmoregulation of maize plants, proline responded significantly to salt stress and to these inoculated strains, while soluble sugars responded less (Fig. 2C). The proline content of maize under salt stress increased 6-fold compared with the control. However, the proline content decreased significantly (*P < *0.05) after inoculation with the strain, which was not significantly different from the control (Fig. 2C). In addition, the two strains reduced the accumulation of malondialdehyde (MDA) and improved root activity of maize (Fig. 2C).

**FIG 3:**
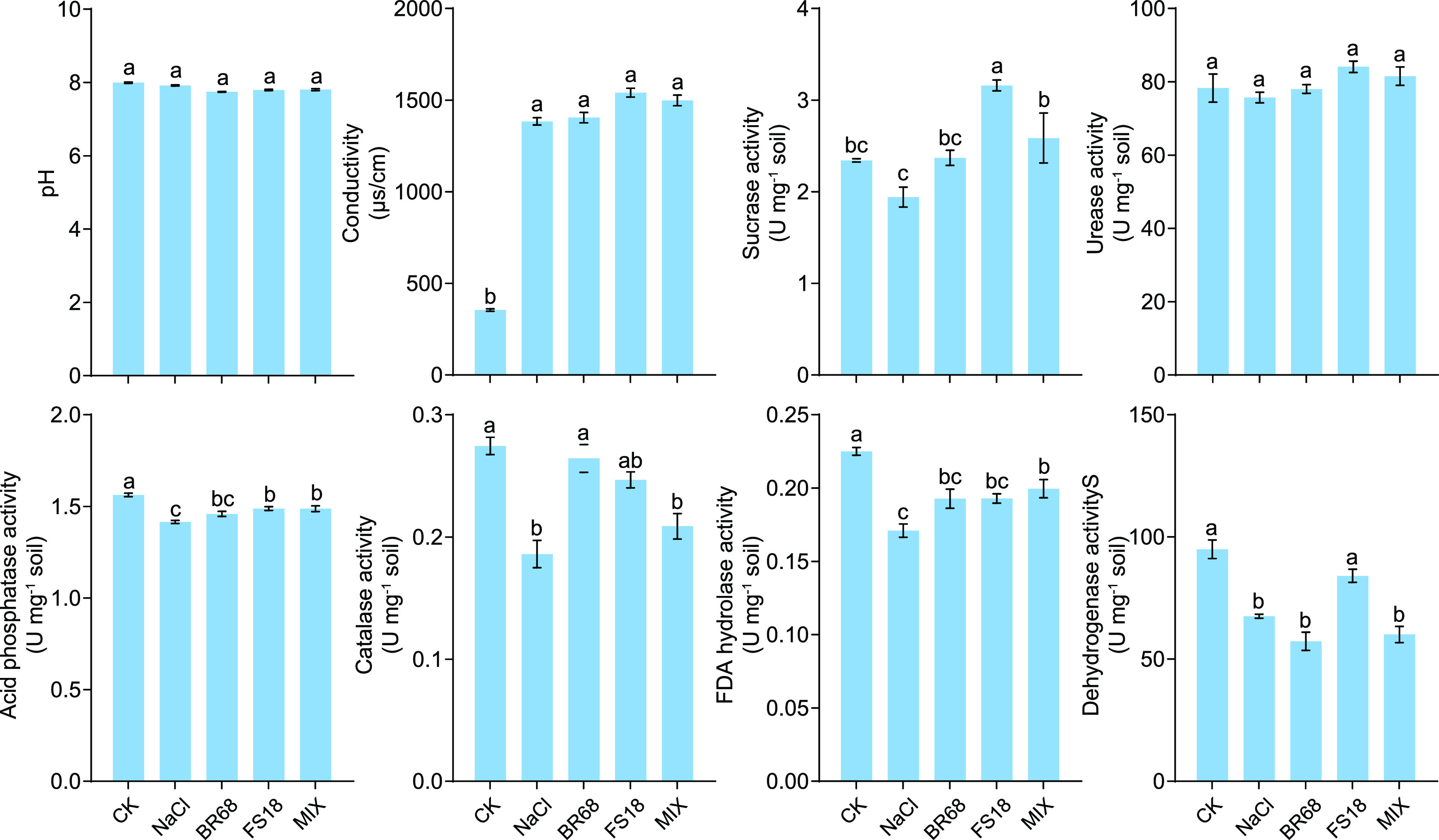
Soil properties under different treatments after 21 days of application. Average ± standard error from three separate replicates. Values with different letters are significantly different at *P* ≤ 0.05 by variance analysis.

In terms of soil properties, these inoculated strains did not affect soil pH and conductivity significantly under salt stress (*P* > 0.05), but some soil enzyme activities were increased (Fig. 3). After inoculation with the strain BR68, catalase activity increased by 41.9% compared with the NaCl treatment and recovered to 87.6% of the control, and sucrase activities and fluorescein diacetate (FDA) hydrolase also increased by 22.0% and 12.8%, respectively. The inoculated strain FS18 significantly enhanced the activities of sucrase, acid phosphatase, and dehydrogenase compared with the NaCl treatment, and the sucrase activity was 34.9% higher than that of the control. Inoculation with a mixture of the two strains also significantly enhanced soil urease, acid phosphatase, and FDA hydrolase activities compared with the NaCl treatment (Fig. 3).

### Effects of inoculated strains on soil microbial community composition and diversity under salt stress.

The box plot based on the Shannon and Chao1 indices showed that the bacterial α-diversity was reduced significantly (*P > *0.05) under salt stress. However, in the fungi, only Chao1 was significantly downregulated, with no significant change in the Shannon index (Fig. S8A). To evaluate the differences in β-diversity, principal coordinate analysis (PCoA) based on the Weighted-UniFrac distance matrix was performed at the amplicon sequence variants (ASVs) level. The results showed that salt stress significantly impacted the bacterial communities while the fungal communities were not affected significantly (Fig. S8B). Comparing the three treatments BR, FS, and MIX with the NaCl treatment separately, the results showed that inoculation with strains BR68 and FS18 did not significantly affect the bacterial community structure (Fig. S9A, B, C), but inoculation with strain FS18 alone significantly changed the fungal community structure (Adonis & Anosim test, *P < *0.05) (Fig. S9E). In addition, both the bacterial communities of the BR treatment and the bacterial and fungal communities of the MIX treatment tended to separate from those of the NaCl treatment (Fig. S9A, C, F).

**FIG 4:**
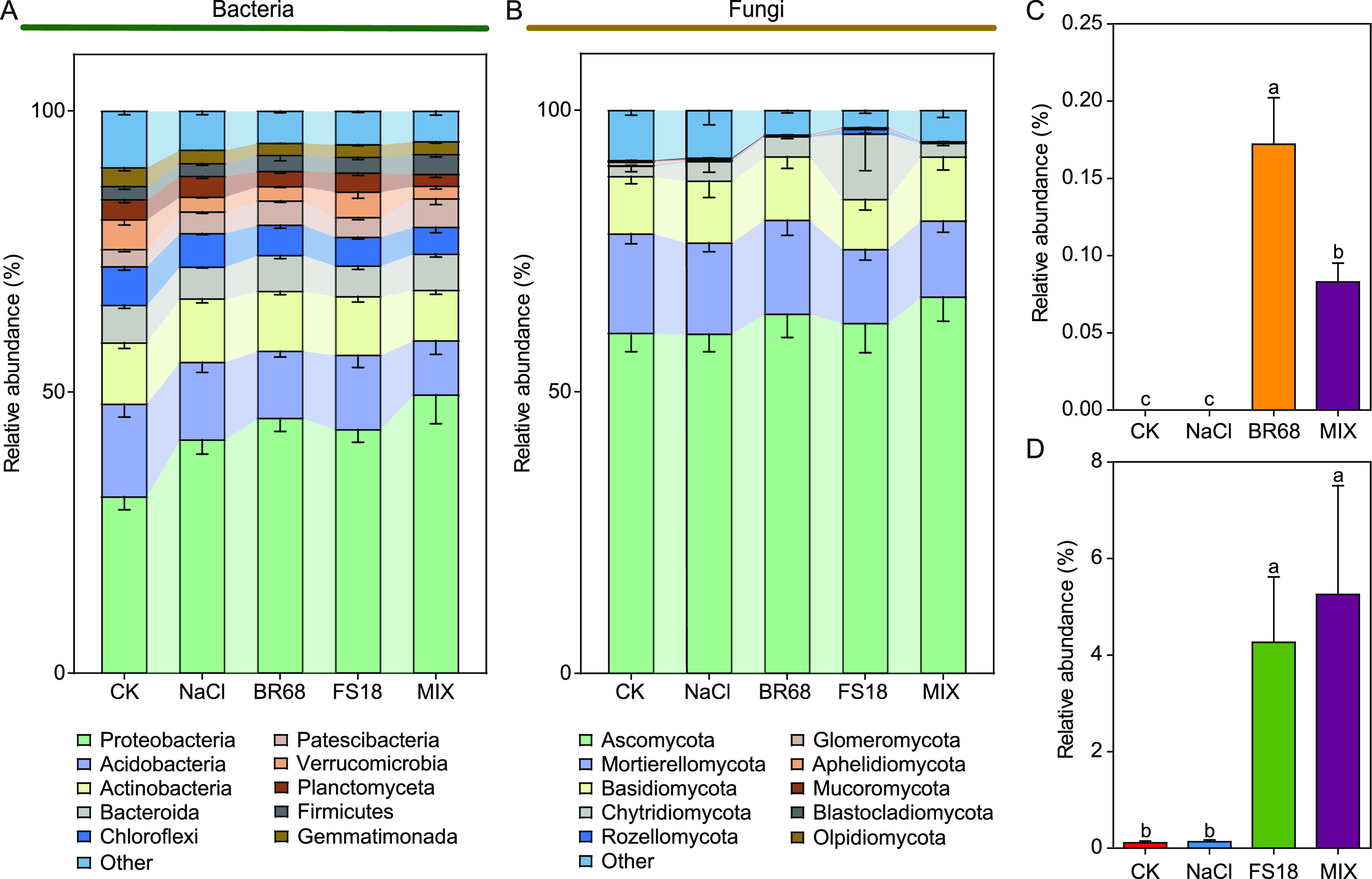
(A) Dominant bacteria (top 10 in relative abundance) and (B) dominant fungi (top 10 in relative abundance) at the phylum level. (C) Relative abundance of inoculated strain BR68 in bacterial communities. (D) Relative abundance of inoculated strain FS18 in fungal communities.

A total of 36 phyla and 602 genera with definite classification statuses were detected in the bacterial communities of all samples. Among the phyla, Proteobacteria had the highest abundance (42.24%), followed by Acidobacteria (13.01%) and Actinobacteria (10.45%) (Fig. 4A). A total of 11 phyla and 143 genera with definite classification statuses were detected in the fungal communities of all samples. At the phylum level, Ascomycota was clearly dominant (62.71%), followed by Mortierellomycota (15.42%) and Basidiomycota (10.54%) (Fig. 4B). In bacterial communities, Proteobacteria was significantly enriched under salt stress compared with the controls, and its enrichment was more pronounced in BR and MIX treatments, while Verrucomicrobia was enriched in the FS treatment (Fig. 4A). For the fungal communities, the abundance of the dominant phyla in soils in the NaCl treatment did not change significantly compared with the control. However, compared with the NaCl treatment, the abundance of Ascomycota increased in BR and MIX treatments, the abundance of Chytridiomycota increased, and the abundance of Basidiomycota decreased in the FS treatment (Fig. 4B).

We also tried to find ASVs with the same sequence as these inoculated strains from high-throughput sequencing results. The results showed that the sequence for Bac_ASV291 in the bacterial communities was exactly the same as that of BR68 (Fig. S10A). Combined with the results of the phylogenetic tree, we concluded that Bac_ASV291 was the strain BR68 (Fig. S10B). Similarly, according to the results from the sequence alignment and phylogenetic tree, we concluded that Fun_ASV12 in the fungal communities was FS18 (Fig. S10C, D). These inoculated strains BR68 and FS18 were barely detectable in the control and NaCl treatments. However, in all treatments inoculated with BR68 and/or FS18, a significant increase in the abundance of the corresponding microorganisms could be detected (Fig. 4C and D).

### Correlation among inoculated strains, specific biomarkers, microbial communities, soil properties, and maize physiologies.

**FIG 5:**
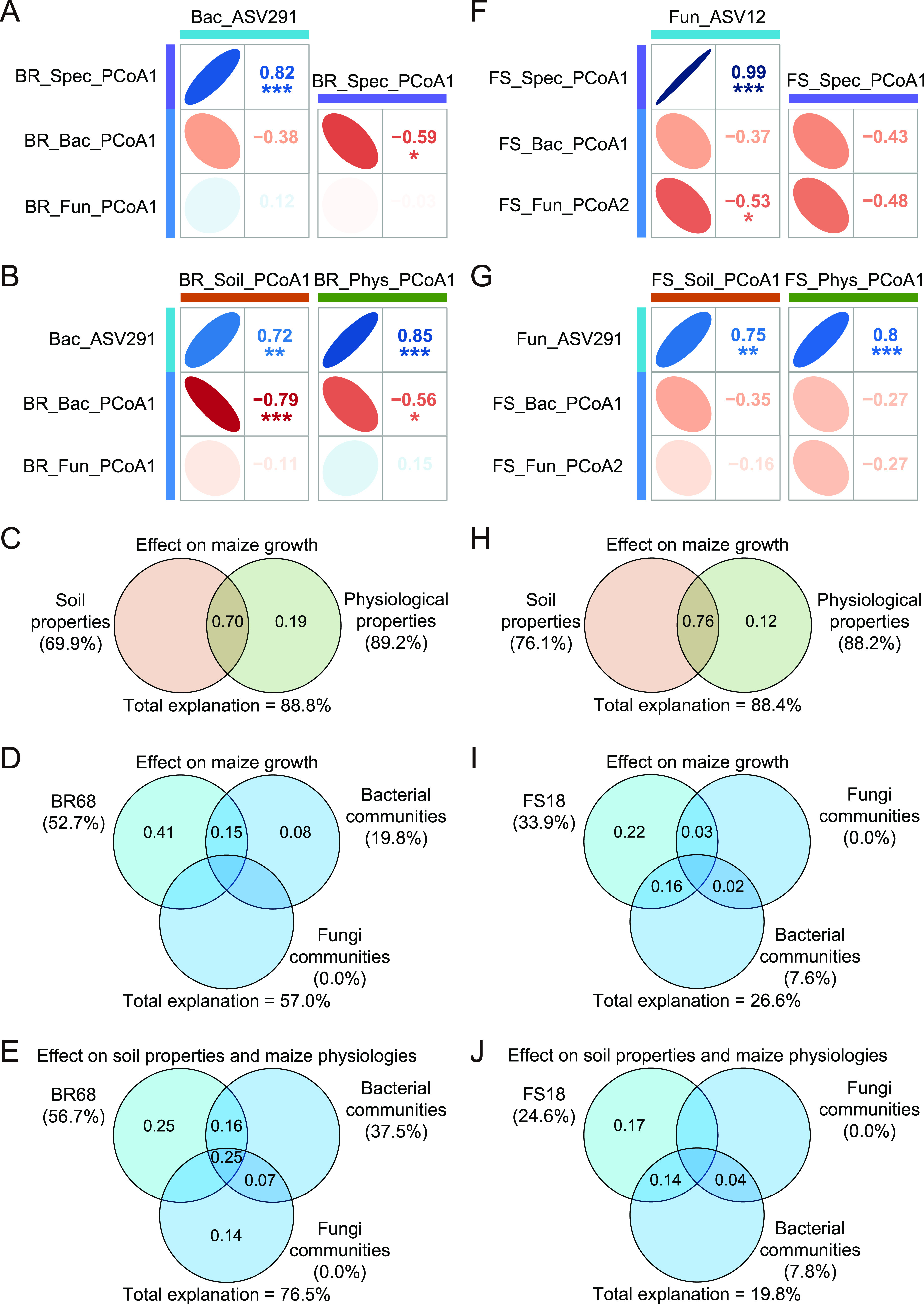
The relationships among inoculated strains, microbial communities, soil properties, and maize physiologies. In Group BR, (A, B) Spearman correlations among the abundance of strain RB68, BR-specific biomarkers, microbial communities, soil properties, and maize physiologies were evaluated; the VPA evaluated (C) the explanatory power of soil properties and maize physiologies to the variation of maize growth, (D) the explanatory power of the abundance of strain RB68, bacteria, and fungal communities to the variation of maize growth, and (E) the explanatory power of the abundance of strain RB68, bacteria, and fungal communities to the variation of soil properties and maize physiologies. In Group FS, (F, G) Spearman correlations among the abundance of strain FS18, FS specific biomarkers, microbial communities, soil properties, and maize physiologies were evaluated; the VPA evaluated (H) the explanatory power of soil properties and maize physiologies to the variation of maize growth, (I) the explanatory power of the abundance of strain FS18, bacteria, and fungal communities to the variation of maize growth, and (J) the explanatory power of the abundance of strain FS18, bacteria, and fungal communities to the variation of soil properties and maize physiologies.

Spearman correlation analyses were conducted to further evaluate relationships among these inoculated strains, specific biomarkers, microbial communities, soil properties, and maize physiologies. In Group BR, the results showed that BR-specific biomarkers (BR_Spec_PCo1) were significantly correlated not only with the abundance of strain BR68 (Bac_ASV291) (*P < *0.05) but also with bacterial communities (Fig. 5A). The variation partitioning analysis (VPA) results further showed that the explanatory power of BR-specific biomarkers for the variation in bacterial communities reached 93.5%, much higher than that of the abundance of strain BR68 (Fig. S11A). The abundance of strain BR68 and bacterial communities were significantly correlated (*P < *0.05) with soil properties (BR_Soil_PCo1) and maize physiologies (BR_Phys_PCo1), respectively (Fig. 5B). Soil properties were also significantly correlated (*P < *0.05) with maize physiologies (Fig. S12A). The VPA showed that the total explanatory power of soil properties and maize physiologies for the variation in maize growth was 88.7%, with maize physiologies having a slightly higher explanatory power (89.2%) than soil properties (69.9%) (Fig. 5C). The total explanatory power of the abundance of strain BR68 and microbial communities for the variation in maize growth was 57.0%, while the explanatory power for the variation in soil properties and maize physiologies reached 76.5% (Fig. 5D and E). In Group FS, the correlation analysis results showed that the abundance of strain FS18 correlated significantly with FS-specific biomarkers and fungal communities, while the correlation with bacterial communities was not significant (Fig. 5F). There were significant correlations among the abundance of strain FS18, soil properties (FS_Soil_PCo1), and maize physiologies (FS_Phys_PCo1) (*P < *0.05) (Fig. 5G). The VPA results showed that the explanatory power of soil properties and maize physiologies for the variation in maize growth was 76.1% and 88.2%, respectively (Fig. 5H). The total explanatory power of the abundance of the strain FS18 and bacterial and fungal communities for the variation in maize growth was 26.6%, and 19.8% for the variation in soil properties and maize physiologies (Fig. 5I and G). In addition, we found that in Group BR, the genera *Vicinamibacter*, *Reyranella*, *Gemmata*, and OM190 were significantly correlated with most soil properties, and the genera *Reyranella*, *Candidatus Chloroploca*, and *Gemmata* were significantly correlated with most maize physiological parameters, but the trends in their correlations were an opposite to that of the abundance of strain BR68 (Fig. S13A). However, in Group FS, the genus *Oligoflexus* was significantly correlated with most soil properties and maize physiologies and showed a consistent correlation trend with the abundance of strain FS18 (Fig. S13B).

### Effect of inoculated strains on microbial communities, soil properties, maize physiologies. and maize growth.

**FIG 6:**
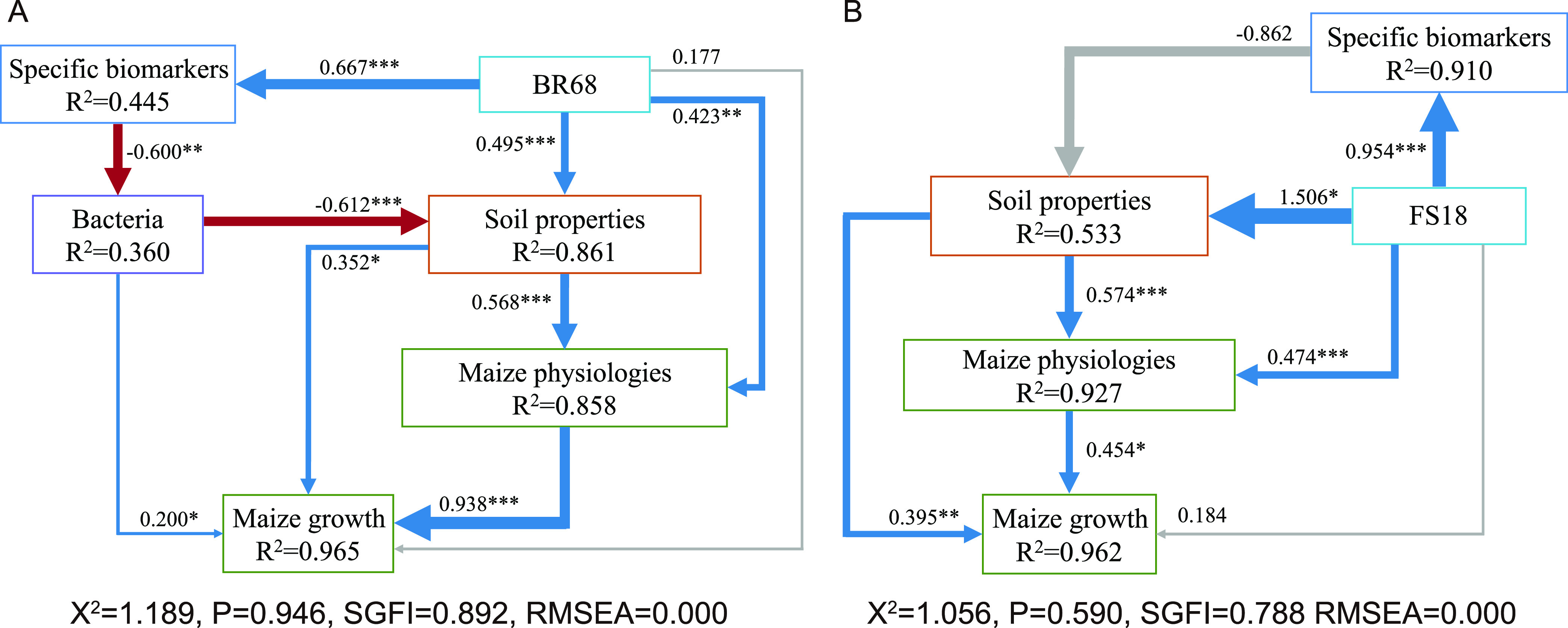
Structural equation modeling (SEM) for Group BR (A) and Group FS (B). The value above the SEM line represents the path coefficient, * represents a significant difference (***, *P < *0.001; **, *P < *0.01; *, *P < *0.05). The blue line represents the positive path coefficient, the red line represents the negative path coefficient, and the black line represents the nonsignificant path coefficient. The width of the arrow indicates the size of the standard path coefficient.

Structural equation modeling (SEM) was used to analyze the direct and indirect effects of inoculated strains on microbial communities, soil properties, maize physiologies, and maize growth in Group BR and Group FS, respectively. In Group BR, the model fit the data well (χ^2^ = 1.189, *P* = 0.946, SGFI = 0.892, RMSEA = 0.000) and explained 44.5% of the variation in BR-specific biomarkers, 36.0% of the variation in bacteria, 86.1% of the variation in soil properties, 85.8% of the variation in maize physiologies, and 96.5% of the variation in maize growth (Fig. 6A). The results showed that the abundance of strain BR68 indirectly influenced bacterial communities (λ = −0.600, *P < *0.01) by directly influencing BR-specific biomarkers (λ = 0.667, *P < *0.001), and thus bacterial communities significantly affected the soil properties (λ = –0.612, *P < *0.001). Soil properties directly influenced maize physiologies (λ = 0.568, *P < *0.001), and maize physiologies directly influenced maize growth (λ = 0.938, *P < *0.001) (Fig. 6A). In addition, the abundance of strain BR68 significantly influenced soil properties (λ = 0.495, *P < *0.001) and maize physiologies (λ = 0.423, *P < *0.01), and maize growth was also directly influenced by bacterial communities (λ = 0.200, *P < *0.05) and soil properties (λ = 0.352, *P < *0.05) (Fig. 6A). In Group FS, the model also fit the data well (χ^2^ = 1.056, *P* = 0.590, SGFI = 0.788, RMSEA = 0.000) and explained 91.0% of the variation in FS-specific biomarkers, 53.3% of the variation in soil properties, 92.7% of the variation in maize physiologies, and 96.2% of the variation in maize growth (Fig. 6B). The results showed that the abundance of strain FS18 significantly influenced soil properties (λ = 1.506, *P < *0.05), which significantly influenced maize physiologies (λ = 0.574, *P < *0.001). Maize growth was directly influenced by the maize physiologies (λ = 0.454, *P < *0.05) and soil properties (λ = 0.395, *P < *0.01). In addition, the abundance of strain FS18 significantly influenced FS-specific biomarkers (λ = 0.954, *P < *0.001), but FS specific biomarkers had no significant effect on soil properties (λ = 0.862, *P > *0.05) (Fig. 6B).

## DISCUSSION

Rhizosphere microorganisms are an important part of the underground environment of plant and can promote plant growth and enhance plant resistance against various environmental stresses (21–23). In previous research, microorganisms have been shown to mitigate plant damage caused by high salinity, drought, disease, and harmful substances (such as heavy metals and phenolic acids) (24–27). In this study, we reported that two strains (the bacterium Providencia vermicola BR68 and the fungus *Sarocladium kiliense* FS18) isolated from the roots and rhizosphere soil of the halophyte *Suaeda salsa*. The two strains stably colonized the maize rhizosphere soil stably under high salinity conditions, improved the soil enzyme activities, and influenced microbial communities. They also affected the physiologies of maize under salt stress, alleviating toxicity due to salt stress in maize and promoting maize growth.

A high salt concentration in the soil prevents the maize root system from absorbing water properly, thereby affecting the transpiration rate and the intercellular CO_2_ concentration, which in turn leads to a reduction in the net photosynthetic rate and to an accumulation of organic matter (28). Our results also indicate that salt stress has a significant negative effect on the biomass and photosynthesis in maize (Fig. 2B). However, both single and mixed applications of the two strains promoted maize growth and increased the maize dry weight and net photosynthetic rate significantly, indicating that the accumulation of organic matter in maize was improved (Fig. 2B). This phenomenon is strong evidence that the salt stress of maize was alleviated, which is inseparable from the improvement in the physiologies of maize after inoculation with the strains. For example, the CAT, POD, and SOD activities of maize were significantly increased after inoculation with the strain BR68, and the strain FS18 significantly increased the CAT, POD, and APX activities, some of which even surpassed those of the control treatment (Fig. 2C). These antioxidant enzymes work synergistically to remove ROS accumulated in maize leaves due to the stress response and alleviate oxidative damage to maize caused by the disruption of ROS homeostasis under salt stress (29–31). This was also directly demonstrated by the measurement of ROS in our experiments (Fig. 2C). Plants produce MDA due to membrane lipid peroxidation under stress conditions, and MDA is used together with proline as an indicator to determine the degree of plant stress (32, 33). Proline is also an important osmoregulatory substance in plants. Our results showed that the inoculation of strains BR68 and FS18 significantly decreased the proline content of maize compared with the NaCl treatment, and the MDA content also decreased, indicating that the osmotic stress and oxidative damage to maize due to stress were alleviated (Fig. 2C). In addition, the VPA results showed that the explanatory power of maize physiologies on the variation in maize growth in Group BR and Group FS reached 89.2% and 88.2%, respectively (Fig. 5C and H). The results of SEM also showed that maize physiologies can directly affect maize growth, and the contribution of physiologies to growth in Group BR and Group FS reached 93.8% and 45.4%, respectively (Fig. 6). These results all indicate that the improvement in maize physiologies due to these inoculated strains played an important role in alleviating damage to maize due to salt stress. Feng et al. (34) found that the activities of superoxide dismutase, catalase, and ascorbate in maize increased after inoculation with photosynthetic bacteria and potassium-enhancing bacteria, which effectively improved the salt tolerance of maize. Li et al. (35) reported that a novel PGPR strain, Kocuria rhizophila, mitigated the deleterious effects of salinity on maize growth and development by regulating plant hormones and nutrient acquisition, thereby maintaining ionic homeostasis and improving photosynthesis. A plant biostimulant (Megafol-Meg) was also found to reduce the impacts of salt stress on maize growth and photosynthetic pigments by reducing the contents of H_2_O_2_, MDA, and total phenolic compounds (TPC) after inoculation (2). Therefore, the changes in physiologies caused by inoculants are one of the reasons for the improved growth of maize under salt stress.

Although a large number of studies have shown that plant physiologies affect plant growth and resistance under salt stress, soil physicochemical properties and rhizosphere microbial communities also play an irreplaceable role in plant resistance to stress (36, 37). Soil microbiome enzymes are critical in organic matter decomposition and nutrient cycling and are frequently used to evaluate the quality and health of soil ecosystems (38). Our results suggest that salt stress reduces the activity of soil microbiome enzymes, especially catalase (Fig. 3), which is more sensitive to salt stress (39). However, inoculation with the strains increased the activities of soil sucrase, urease, and acid phosphatase (Fig. 3). These enzymes could promote the metabolic cycling of soil nutrients and enhance soil fertility (40). Catalase and FDA hydrolase are important indicators for evaluating the activity and health of soil microorganisms (41, 42). Our experimental results showed that under salt stress, these inoculated strains could improve the activities of catalase and FDA hydrolase (Fig. 3). The increase in these enzyme activities is also a reflection of improved soil microbial activity (43).

In our high-throughput data results, ASVs identical to the 16S sequence of the inoculated strain BR68 and to the ITS sequence of FS18 were detected for the bacterial and fungal results, respectively (Fig. S10). Their abundance increased with the inoculation of the strains (Fig. 4C and D). This indicates that the two strains colonized the maize rhizosphere soil, which indicates that they could function stably. In addition, the changes in soil pH, conductivity, and microbial communities were not significant during inoculation with the bacteria BR68 and fungi FS18, indicating that the strains had little effect on the overall environment of the soil (Fig. 3, Fig. S9). However, the results of the differential analysis showed that specific microorganisms responded to these inoculated strains, and some even improved soil properties and maize physiologies together with these inoculated strains. For example, the genus *Oligoflexus* in the fungal data was significantly enriched in both FS and MIX treatments (Fig. S1B, C). It was significantly and positively correlated with the antioxidant enzyme activity and soil enzyme activity of maize and significantly and negatively correlated with ROS and MDA, which is consistent with the trends in the changes to the maize physiological and soil properties after inoculation with strain FS18 (Fig. 2C and 3, Fig. S13). In addition, the abundance of the genus *Oligoflexus* was significantly and positively correlated with the inoculated strain FS18 (Fig. S3), so we speculate that this genus and the inoculated strain had a synergistic effect in helping maize resist salt stress. Interestingly, we found that the genera *Reyranella* and *Gemmata* in the bacterial data were significantly enriched in the NaCl treatment and significantly and negatively correlated with strain BR68 (Fig. S1A, C, S2). The two genera were also significantly and negatively correlated with most plant- and soil-related enzyme activities and positively correlated with ROS and proline content, so we speculate that the two genera have a negative effect on maize under salt stress, but inoculation with the strain BR68 could inhibit their activity and thus indirectly alleviate the stress damage to the plant. This may be another mechanism by which these inoculated strains enhanced resistance to salt stress in maize. A similar mode of action has been reported for biological control processes. During the development of pathogen infections in plants, plants can recruit specific microorganisms to help plants defend against pathogens, and pathogens can also recruit helpers to increase the activity and infectivity of the pathogens (44–46). However, under salt stress in maize, the mechanism of enrichment and synergy among microorganisms after inoculation with microbial strains has not been fully studied. We also noticed that the inoculated strain affected the relationship among soil microbes, soil physicochemical properties, and maize physiologies. There were significant correlations among the abundance of these inoculated strains, soil properties, and maize physiologies (Fig. 5B and G, Fig. S12). The SEM results also showed that soil properties directly affected maize physiologies and maize growth, and bacterial communities in Group BR contributed to both soil properties and maize growth (Fig. 6). Therefore, the changes in soil properties and soil microorganisms caused by inoculation are also important factors in improving maize physiologies, growth, and salt stress resistance.

Although the two strains significantly improved the salt tolerance of maize, we found differences in the way they worked. In Group BR, although the inoculated strain and microbial communities had a lower explanatory power for the variation of maize growth (Fig. 5D), it could explain 76.5% of the variation in soil properties and maize physiologies (Fig. 5E), indicating that soil microorganisms could indirectly improve maize growth by maize physiologies and soil properties. However, in Group FS, the explanatory power of the variation in soil properties and maize physiologies and the variation in maize growth were mainly contributed by the fungus FS18, with relatively low explanations by bacterial and fungal communities (Fig. 5I and J). In addition, in Group BR, it could be seen from the SEM results that the bacteria BR68 was indirectly associated with bacterial communities by affecting BR specific biomarkers, and bacterial communities significantly contributed to soil enzyme activities and maize growth (Fig. 6A). In the FS group, the fungus FS18 was directly associated with soil properties and maize physiologies (Fig. 6B). The above results implied that BR68 is indirectly associated with bacterial communities through BR-specific biomarkers and directly associated with soil properties and maize physiologies, and promote maize growth under salt stress. The fungus FS18 improves maize growth under salt stress through soil properties and maize physiologies.

We demonstrated that two salt-tolerant microorganisms originating from the rhizospheres of halophytes could improve maize physiologies by changing soil microbial and physicochemical properties, which promoted maize growth and tolerance to salt stress. However, BR68 and FS18 worked in different ways in the soil. This study did not involve a detailed elaboration of the molecular pathways for salt tolerance in maize after inoculation, which may be explored in our future research work.

## MATERIALS AND METHODS

### Isolation and identification of salt-tolerant microorganisms.

Soil samples were collected from the rhizosphere soil of *Suaeda salsa* from Kenli County, Shandong Province (37°12'26"N, 118°48'59"E, North China) and stored at 4°C. One hundred microliters of soil solutions serially diluted 10^3^ to 10^6^-fold with sterile water was spread over plates containing a Luria Bertani (LB) medium with 850 mM NaCl and incubated at 28°C. Different strains were selected from each plate and purified by a streaking method on a LB medium with 850 mM NaCl. After purification, the strains were stored at 4°C using glycerol preservation.

For morphological identification, strain morphologies were observed by the Gram staining and negative staining methods. For molecular identification, the 16S rRNA gene was amplified by the universal primers 27F (5′-AGA GTT TGA TCC TGG CTC AG-3′) and 1492R (5′-GGT TAC CTT GTT ACG ACT T-3′). The final volume of the amplification reaction solution was 25 μL containing 18.35 μL of ddH_2_O, 2.5 μL of 10× buffer, 2 μL of dNTP, 0.5 μL of each primer, 0.15 μL of *Taq* polymerase, and 1 μL of a template solution. The conditions of the PCR were as follows: initial denaturation at 95°C for 10 min, then 35 cycles containing thermal denaturation at 95°C for 40 s, annealing at 53°C for 40 s, extension at 72°C for 90 s, and the final extension at 72°C for 10 min. The obtained 16S rRNA gene sequences were compared with the NCBI database (https://www.ncbi.nlm.nih.gov/). The sequence that matched was combined with the obtained sequence, and a phylogenetic tree was constructed by multiple alignments using the Molecular Evolutionary Genetics Analysis (MEGA) software (version 7.0) and the neighbor-joining method (47). The strain was identified based on a 16S rRNA gene sequence analysis, combined with morphological and biochemical properties referenced from *Bergey’s Manual of Determinative Bacteriology* (48).

### Pot experiment.

To obtain bacterial suspensions of BR68 and FS18, they were cultured in an LB medium supplemented with 200 mM NaCl and incubated at 28°C for 3–4 days. The absorbance of the bacterial suspensions was measured by a spectrophotometer and adjusted to 0.8 with a sterile LB medium supplemented with 200 mM NaCl.

Maize was planted in a medium consisting of common agricultural soil and vermiculite at a ratio of 1:1. Maize seedlings were divided into five groups (10 pots per group): control (nonsaline soil); NaCl treatment (200 mM NaCl); BR68 treatment (200 mM NaCl + Providencia vermicola BR68); FS18 treatment (200 mM NaCl + *Sarocladium kiliense* FS18); and MIX treatment (200 mM NaCl + Providencia vermicola BR68 + *Sarocladium kiliense* FS18; the total amount of inoculum of these two strains was the same as the amount of inoculum in the above treatments). Each seed of maize was germinated at 28°C for 2 days and planted in a 25-cm-diameter plastic pot containing 1,500 g of the mixed soil. They were cultured at 25°C with a photoperiod of 16 h of light (600 μmol m^−2^ s^−1^)/8 h of darkness and watered with a half-strength Hoagland’s solution twice a week (49). All treatments were applied after the maize seeds germinated in the soil, and bacterial suspensions of BR68 and FS18 were applied to the soil in which maize was planted by irrigating the roots.

### Sampling and determination of maize growth, physiologies, and soil properties.

We chose to sample near the end of the maize seedling stage. Since it takes 6–7 days for germination, we chose to sample at 21 days after strain inoculation. First, the plant height, root length, and fresh weight of maize were determined. Net photosynthesis rate (Pn) was determined using a portable gas exchange analyzer (LI-6400 XT, LI-COR, NE, USA) at 30°C during the 9:30–11:30 period at a CO_2_ concentration of 380 μmol mol–1 and at 37–42% relative humidity (50). Three maize seedlings were randomly selected from each pot, and the dry weights were determined after drying to a constant weight at 70°C. Then the leaves from the remaining maize seedlings were used to determine maize physiologies after the veins were removed, and the roots were used to determine root activity. In addition, the soil within 2 mm of the maize root was collected, the maize root tissue and debris were removed from the soil, and the soil was stored as the rhizosphere soil at 4°C and −80°C for determining the soil enzyme activities and microbial communities, respectively. Each sample used to determine biomass, physiology, soil properties, and microbial communities was in parallel and in one-to-one correspondence to adapt to the subsequent correlation analysis.

Maize leaf samples (0.5 g) were ground with a mortar and pestle on ice, and 10 mL of a 50-mM sodium phosphate buffer (pH 7.8) was added to form a homogenate. The homogenate was centrifuged at 10,000 g for 10 min at 4°C. The supernatant was collected as an enzyme extract and used to analyze for the rate of active oxygen generation, soluble proteins (51), and antioxidant enzyme activities. The enzymatic activities of SOD, CAT, APX, and POD were determined by the nitro-blue tetrazolium (NBT) photoreduction method (52), the hydrogen peroxide decomposition method (53), the ascorbic acid method (54), and the guaiacol method (55), respectively. Soluble sugar content in maize leaves was measured by the anthrone method (56). Proline content was determined according to the method of Bates et al. (57). To determine the extent of NaCl-induced oxidative stress, lipid peroxidation was evaluated by measuring the MDA content of maize leaves according to the method of Demiral and Turkan (58). Root activity was measured through the triphenyl tetrazolium chloride (TTC) method (59).

Soil and water were mixed at a ratio of 1:2.5 and centrifuged at 4,000 r/min for 10 min. The supernatant was used to determine pH and conductivity using a pH electrode and a conductivity meter (Leici, Shanghai, China), respectively. Soil sucrase, urease, and catalase activities were determined by the 3,5-dinitrosalicylic acid colorimetric method, indophenol colorimetric method, and volumetric method, respectively (60–62). The sucrase activity was expressed as the number of milligrams of glucose produced per gram of soil after 24 h, and the urease activity was expressed as the number of milligrams of NH3-N released by urea hydrolysis per gram of soil at 37°C for 24 h (63). Acid phosphatase activity was measured at 25.5°C using an acetate buffer (pH 4.50) according to the method of Pawar and Thaker (64). The dehydrogenase activity was determined using the 2,3,5-triphenyl tetrazolium chloride (TTC) reduction method (65). An enzymatic unit was defined as the amount of TPF per gram of the soil sample produced in 24 h. The FDA hydrolase activity was determined according to the method of Adam and Duncan (41).

### DNA extraction, Illumina MiSeq sequencing and analysis.

Rhizosphere soil DNA was extracted using the E.Z.N.A. Soil DNA Kit (Omega, USA) according to the manufacturer’s instructions, and the quantity and quality of DNA was determined by a NanoDrop 2000 spectrophotometer (Thermo Scientific, USA). Aliquots of the DNA were stored at −80°C.

The V3–V4 regions of the bacterial 16S rRNA gene were amplified using the primers 515F (5′-GTGCCAGCMGCCGCGGTAA-3′) and 806R (5′-GGACTACHVGGGTWTCTAAT-3′). The primers ITS1F (5′-CTTGGTCATTTAGAGGAAGTAA-3′) and ITS2R (5′-GCTGCGTTCTTCATCGATGC-3′) were used to amplify the ITS1–ITS2 region of the fungal internally transcribed spacer. Amplicon libraries were constructed using the NEB Next Ultra DNA Library Prep Kit from Illumina (NEB, USA). The amplicons were sequenced on an Illumina MiSeq platform (Majorbio Bio-Pharm Technology Company, China).

The quality of the raw sequence was evaluated and low-quality cut-offs for forward and reverse readings were determined. QIIME2 (v. 2020.8) was used to perform quality control and generate an amplicon sequence variant (ASV) feature table (66). The quality control function in DADA2 was used for noise cancellation, chimera detection, and removal (67). In addition, we removed ASVs that were present in only 1 sample or with an abundance of less than 5. The ASV representative sequences were aligned with the SILVA database 138 (http://www.arb-silva.de/). ASV sequences present in only one sample or with an abundance of less than 5 were removed. To accurately assess the diversity of the microbial communities, all samples were rarefied to the same depth based on the minimum sequence number. Sequence numbers were normalized to 68,927 for all samples of bacteria and to 53,704 for all samples of fungi. The subsequent analyses conducted in this study were based on normalized data.

### Definition of specific biomarkers.

Linear discriminant analysis (LDA) effect size (LEfSe) analysis was used to identify biomarkers in the different treatments on the online Galaxy (http://huttenhower.sph.harvard.edu/galaxy/) platform with an LDA score of >2. We found 20, 15, and 32 genera that were significantly different in abundance between the BR68 and NaCl treatments, FS18 and NaCl treatments, and MIX and NaCl treatments, respectively (Fig. S1). We combined the differential genera obtained from the analysis between the BR and NaCl treatments with the differential genera obtained from the analysis between the MIX and NaCl treatments as biomarkers for significant variation in abundance after inoculation with strain BR68 (Fig. S1A, C). We evaluated the correlation of biomarkers with Bac_ASV291 using Spearman correlation analysis and found that 12 biomarkers were significantly correlated with Bac_ASV291 (10 bacteria and 2 fungi) (Fig. S2). The 12 biomarkers were named as the specific biomarkers related to the strain BR68 (BR-specific biomarkers). Similarly, the abundant genera that were different between the FS and NaCl treatments were combined with those between the MIX and NaCl treatments for correlation analysis with Fun_ASV12, and 16 biomarkers were screened (14 bacteria and 2 fungi) (Fig. S1B, C, S3). The 16 biomarkers were named as the specific biomarkers related to the strain FS18 (FS-specific biomarkers).

To more precisely analyze the interaction of each strain with the maize seedlings and soil microbial communities under salt stress, we divided the four treatments NaCl, BR, FS, and MIX into two groups for downstream analysis. We combined the three treatments related to the inoculated strain BR68, namely, NaCl, BR, and MIX treatments, and labeled the group as Group BR. Similarly, we combined the three treatments related to the inoculated strain FS18, namely, NaCl, FS, and MIX treatments, and labeled the group as Group FS. The distance matrices for the fungal and bacterial communities in this study were calculated using QIIME (http://qiime.org/) software with the Weighted-Unifrac metric, and principal coordinate analysis (PCoA) was performed using the prcomp function in the R package “stats.” The distance matrices for specific biomarkers, soil properties, maize physiologies, and maize growth were calculated using the R package “vegan” (v. 2.5-7) with the Bray-Curtis metric. Before calculating the distance matrix, the maize growth and physiologies and soil properties were standardized by logarithmic transformation (lg X) (shi). Here, we chose the PCo1 axis of Fig. S4 to represent the main characteristics of the bacterial and fungal communities of Group BR (labeled BR_Bac_PCo1 and BR_Fun_PCo1) and chose the PCo1 axis of Fig. S5 to represent the main characteristics of the bacterial and fungal communities of Group FS (labeled FS_Bac_PCo1 and FS_Fun_PCo1). In addition, we performed PCoA based on Bray-Curtis distances for specific biomarkers, soil properties, and maize physiologies of Group BR and Group FS, respectively. We extracted their PCo1 axes separately to represent the main characteristics of these indicators (Group BR was labeled BR_Spec_PCo1, BR_Soil_PCo1, and BR_Phys_PCo1, and Group FS was labeled FS_Spec_PCo1, FS_Soil_PCo1, and FS_Phys_PCo1). These PCo1 axes have been validated to have significant correlations with most of their respective indicators (Fig. S6, S7).

### Statistical analysis.

Statistically significant differences among treatments in maize growth, physiologies, and soil properties were evaluated by one-way analysis of variance (ANOVA) using statistics from the base R package “stats” (v. 4.0.3). α-Diversity (including Chao 1, Shannon, Simpson, and Ace) was calculated using the Mothur pipeline (v. 1.34.4) (68). The Spearman correlation coefficients among microorganisms, maize physiology, and soil properties were calculated and plotted using the R package “corrplot” (v. 0.88). Variation partitioning analysis (VPA) was performed by the R package “vegan,” and the adjusted R-squared value was used to evaluate the partitioning between explanatory variables and a response variable. The structural equation model (SEM) was developed by AMOS (IBM SPSS Amos 23) with maximum-likelihood estimation. The model was used to evaluate the indirect and direct effects of inoculated strains on microbial communities, soil properties, maize physiologies, and maize growth. The SEM fitness was examined based on a nonsignificant chi-squared test (*P > *0.05), and the root mean square error of approximation (RMSEA). Bacteria, specific biomarkers, soil properties, maize physiologies, and maize growth represented the data for the first PCoA axis based on their corresponding distances.

### Data availability.

High-throughput sequencing data of 50 samples (including 25 bacterial communities and 25 fungal communities) have been stored in the NCBI database with accession number PRJNA819257.
