# Supplementary figures and images for: Suaeda salsa Root-Associated Microorganisms Could Effectively Improve Maize Growth and Resistance under Salt Stress
Source: Microbiol Spectr. 2022 Aug 11;10(4):e01349-22. doi: 10.1128/spectrum.01349-22 (PMC9430135; doi:10.1128/spectrum.01349-22)

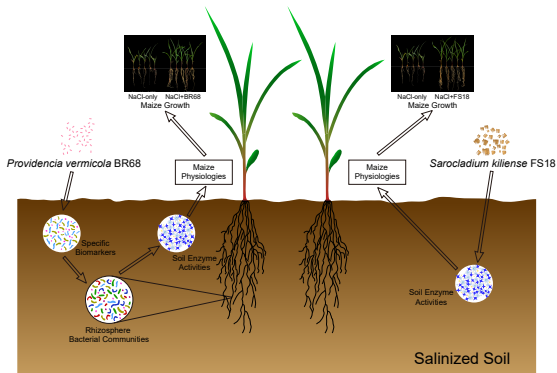

Supplement: Supplemental file 1 — Supplemental material. Download spectrum.01349-22-s0001.pdf, PDF file, 4.7 MB [file spectrum.01349-22-s0001.pdf]
