# Supplementary material for: Suaeda salsa Root-Associated Microorganisms Could Effectively Improve Maize Growth and Resistance under Salt Stress
Source: Microbiol Spectr. 2022 Aug 11;10(4):e01349-22. doi: 10.1128/spectrum.01349-22 (PMC9430135; doi:10.1128/spectrum.01349-22)
Supplement: Supplemental file 2 — Supplemental material. Download spectrum.01349-22-s0002.pdf, PDF file, 1.7 MB [file spectrum.01349-22-s0002.pdf]

Table S1 Preliminary test results of the soilless culture experiment

Effect Type	Strain	Root Length	Plant Height
Control	NaCl	14.51±1.89	10.17±0.83
No Effect	BR11	14.25±0.89	11.13±0.99
	BR12	15.21±1.30	11.35±1.08
	BR15	13.94±0.59	10.01±0.34
	BR17	13.69±0.81	10.97±0.44
	BR22	15.56±0.66	11.09±1.18
	BR25	14.62±0.19	10.87±0.86
	BR27	16.98±0.86	11.66±1.42
	BR33	15.51±0.77	11.05±0.29
	FR4	17.01±1.97	11.55±0.65
	FR5	15.26±0.37	10.92±0.58
	FR7	15.67±0.16	11.35±0.94
	BS43	15.68±1.39	9.67±0.47
	BS44	13.61±0.88	11.03±0.34
	BS54	16.68±0.33	11.76±0.53
	BS62	13.79±0.51	10.10±0.71
	FS16	14.82±0.61	11.02±0.22
	FS19	16.62±0.99	11.79±0.58
Inhibition	BR14	12.18±0.81	6.12±0.57
	BR16	11.54±0.56	5.84±0.83
	FR16	9.58±1.52	6.10±0.21
	BS7	11.22±0.94	6.54±0.65
	BS12	10.23±0.17	7.96±1.41
	BS46	10.62±0.76	5.15±1.03
	BS48	10.89±0.59	6.18±0.48
	FS11	12.02±0.55	7.26±0.57
	FS14	9.61±0.89	5.01±0.66
	FS15	11.68±0.78	6.09±0.72
Promotion	BR23	22.34±1.76	13.05±0.72
	BR68	24.15±0.89	13.52±0.67
	BR76	21.38±0.39	12.03±0.62
	FS18	25.11±0.39	14.52±0.19
	BS47	20.44±0.95	13.07±0.60
	BS66	22.56±0.77	12.18±0.82

* All pot pre-experiments were carried out under the soil with 200mM salt concentration. “No Effect” represents that root length or plant height was not significantly different from control. “Inhibition” represents that root length and plant height were significantly lower than control. “Promotion” represents root length and plant height were significantly higher than control.

Table S2 Characteristics of BR68 and FS18

Characteristics	BR68	FS18
Morphology	Short rod	
Colony pigmentation	milky white	
Acid from glucose	+	+
Oxidase	-	+
Production of indole acetic acid	-	-
Sea-salt range (%, w/v)	0.1-10	0.1-20
Sea-salt optimum (%, w/v)	5	5
Temperature range (uC)	10-45	15-35
Hydrolysis of Starch	+	-
Hydrolysis of Gelatin	-	-
Hydrolysis of Tyrosine	+	-

*“-” represents negative;“+” represents positive.

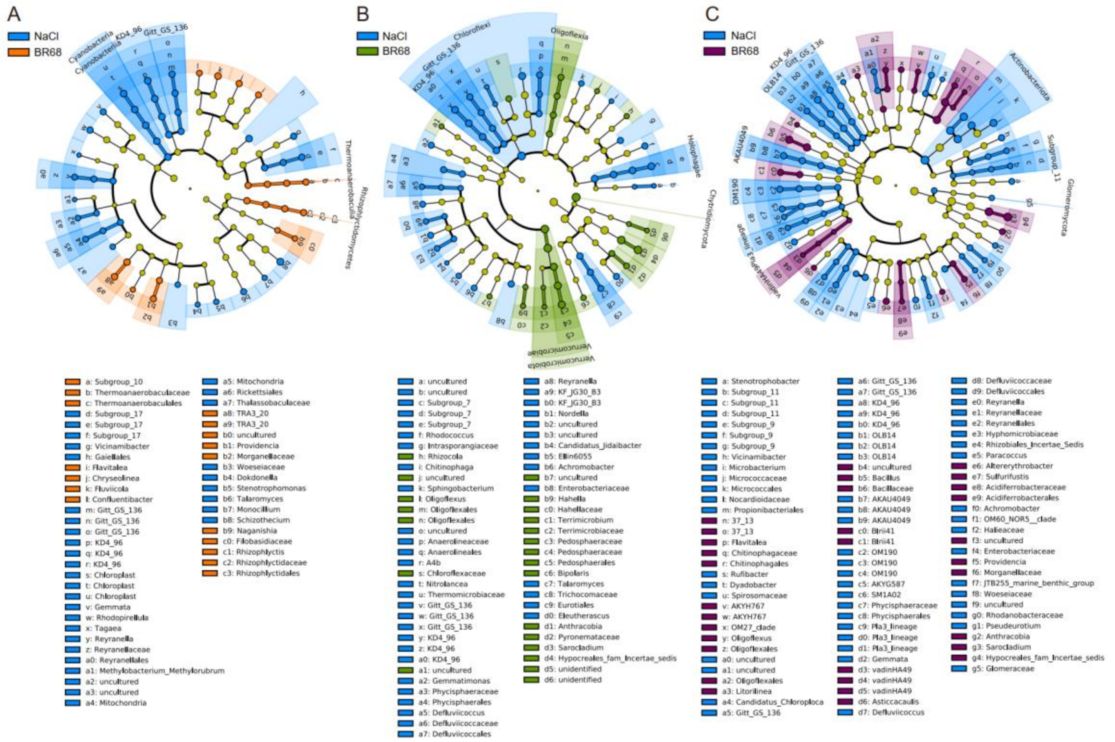

Fig. S1 Significantly ($P < 0.05$) differential biomarkers between (A) BR68 and NaCl treatments, (B) FS18 and NaCl treatments, (C) MIX and NaCl treatments evaluated by the linear discriminant analysis effect size (LEfSe) with LDA scores > 2.

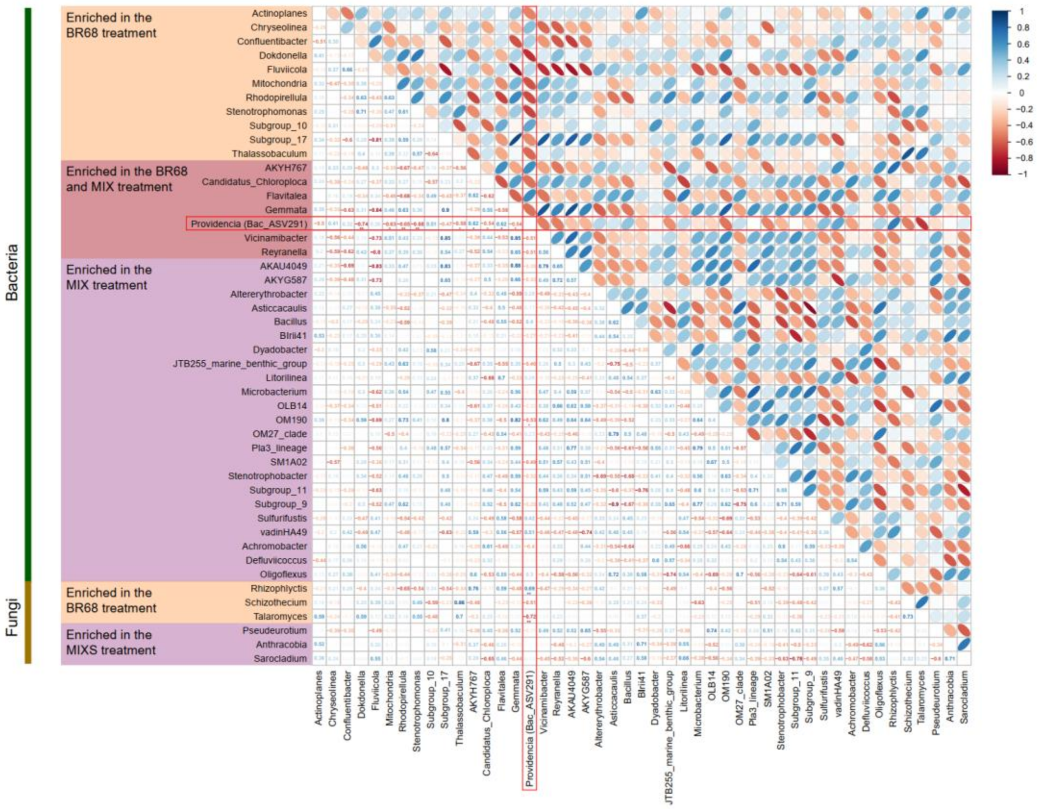

Fig. S2 Spearman correlation between the abundance of strain BR68 and biomarkers of significant variation in abundance after inoculation with strain BR68 in Group BR.

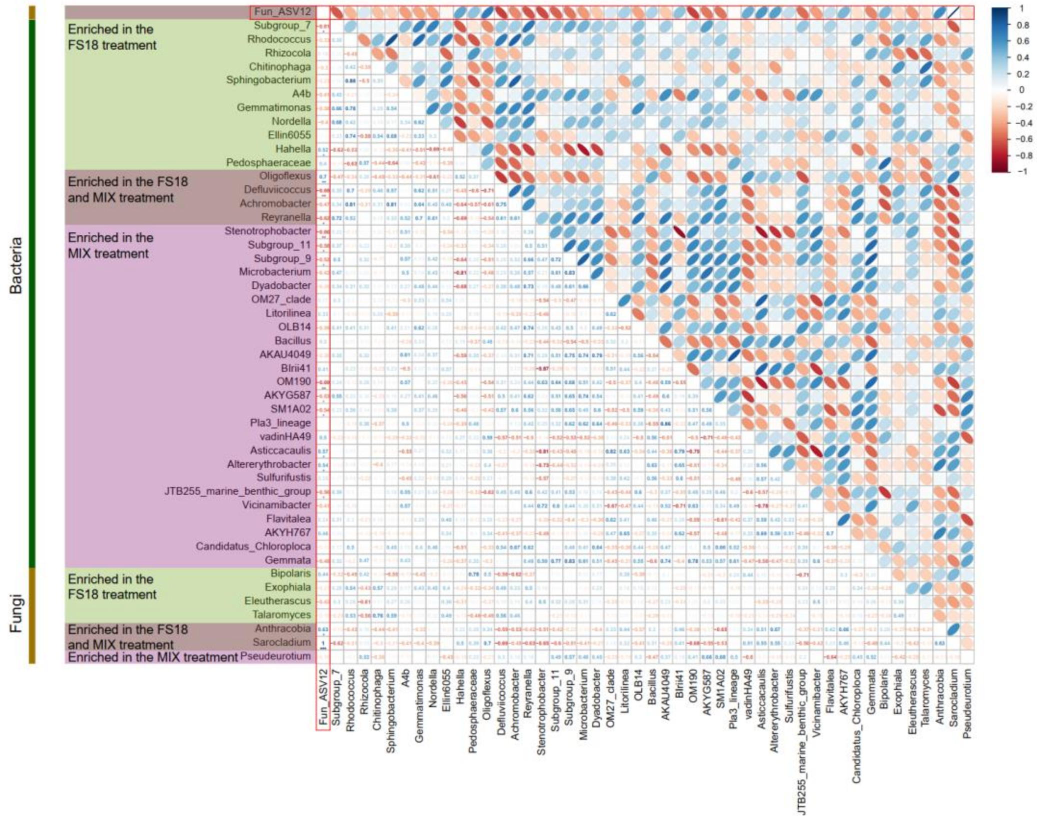

Fig. S3 Spearman correlation between the abundance of strain FS18 and biomarkers of significant variation in abundance after inoculation with strain FS18 in Group FS.

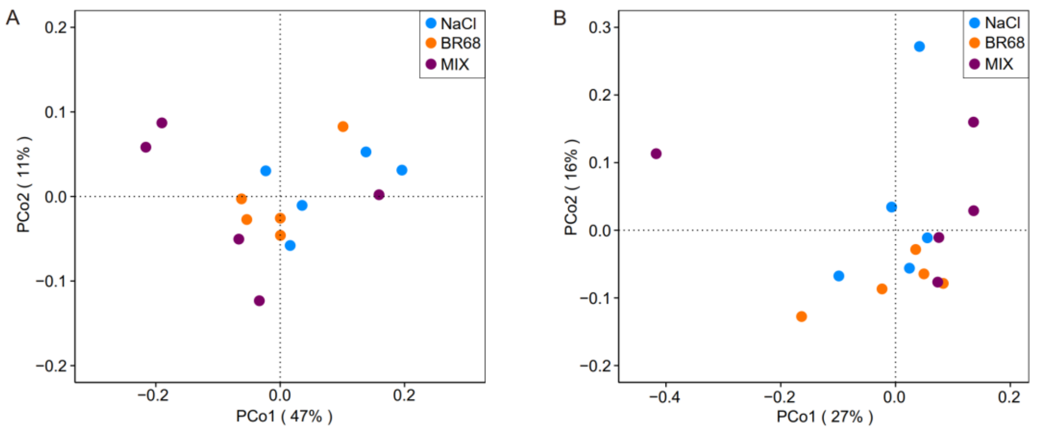

Fig. S4 The principal coordinates analysis (PCoA) based on Weighted-Unifrac distance of (A) bacterial community and (B) fungal community compositions in Group BR.

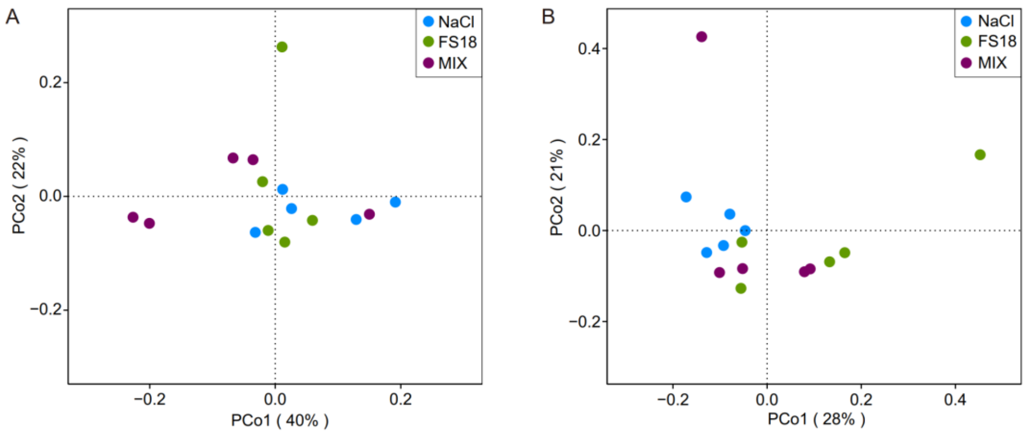

Fig. S5 The principal coordinates analysis (PCoA) based on Weighted-Unifrac distance of (A) bacterial community and (B) fungal community compositions in Group FS.

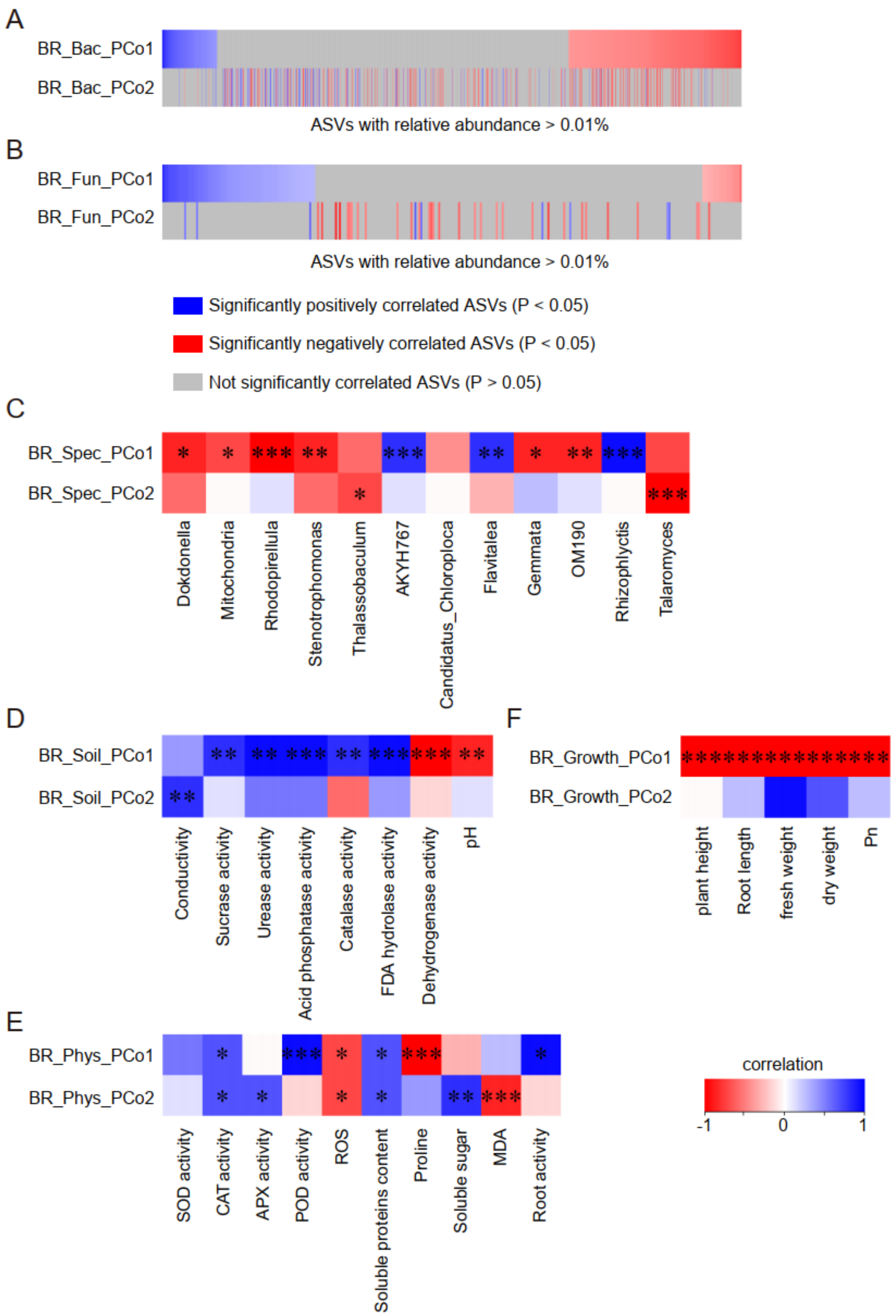

Fig. S6 In Group BR, Spearman correlation (A) between BR-Bac-PCo1/2 and bacterial communities at the OTU level, (B) between BR-Fun-PCo1/2 and fungal communities at the OTU level, (C) between BR-Spec-PCo1/2 and specific biomarkers, (D) between BR-Soil-PCo1/2 and soil properties, (E) between BR-Phys-PCo1/2 and maize physiologies, and (F) between BR-Growth-PCo1/2 and maize growth.

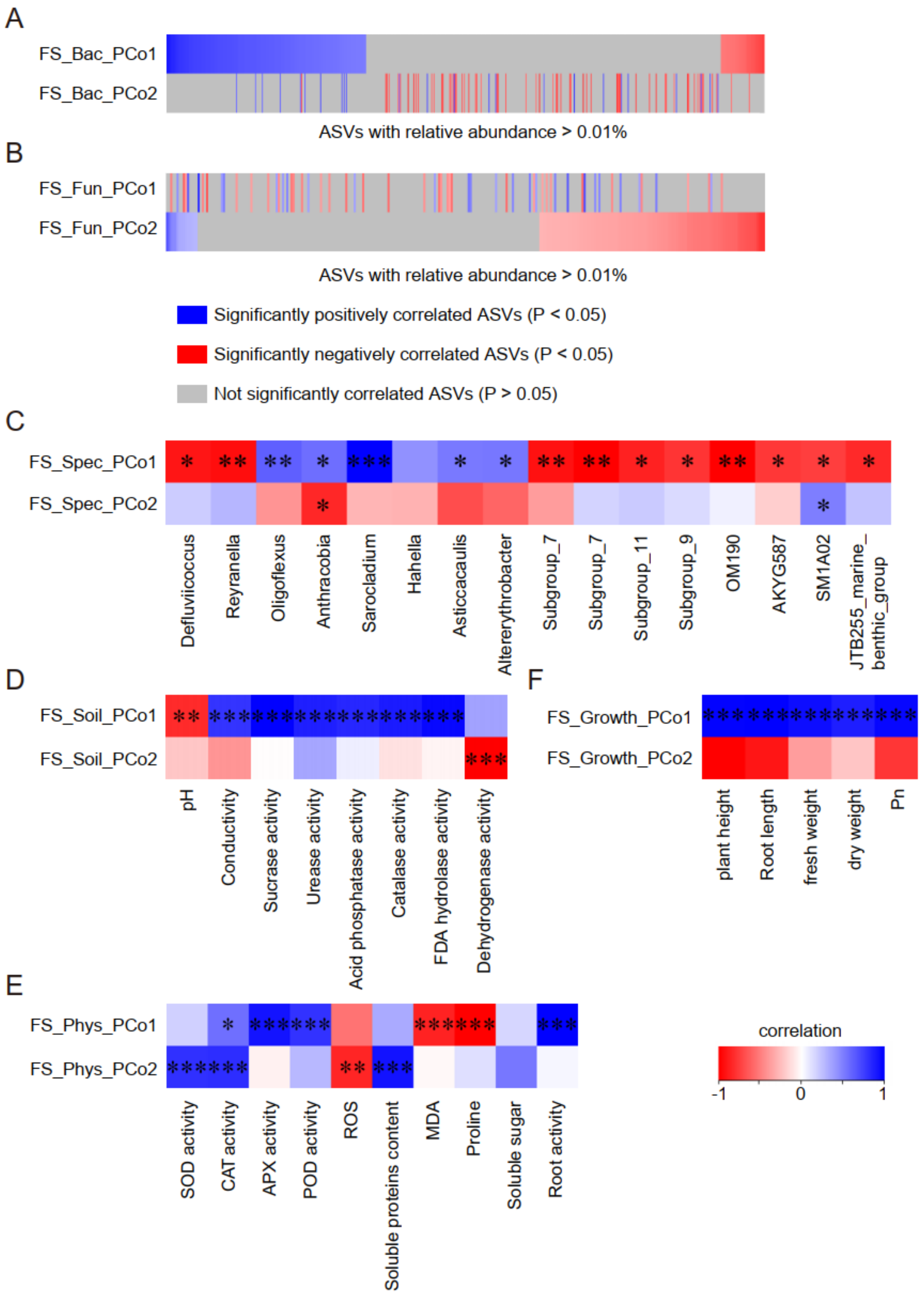

Fig. S7 In Group FS, Spearman correlation (A) between FS-Bac-PCo1/2 and bacterial communities at the OTU level, (B) between FS-Fun-PCo1/2 and fungal communities at the OTU level, (C) between FS-Spec-PCo1/2 and specific biomarkers, (D) between FS-Soil-PCo1/2 and soil properties, (E) between FS-Phys-PCo1/2 and maize physiologies, and (F) between FS-Growth-PCo1/2 and maize growth.

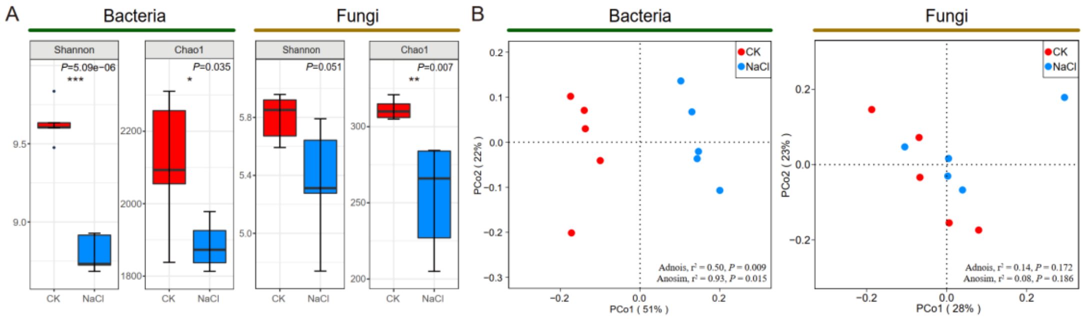

Fig. S8 Shannon and Chao1 indexes of bacterial and fungal communities between control and NaCl treatment.

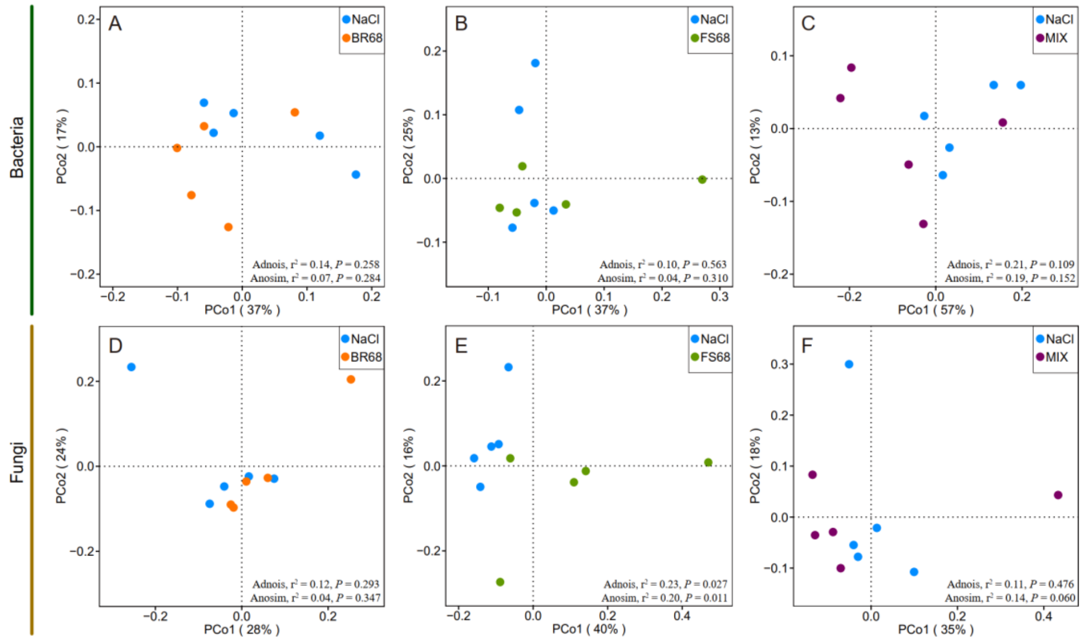

Fig. S9 The principal coordinates analysis (PCoA) of bacterial communities based on Weighted-Unifrac distance of (A) BR68 and NaCl treatments, (B) FS18 and NaCl treatments, (C) MIX and NaCl treatments. The principal coordinates analysis (PCoA) of fungal communities based on Weighted-Unifrac distance of (D) BR68 and NaCl treatments, (E) FS18 and NaCl treatments, (F) MIX and NaCl treatments.

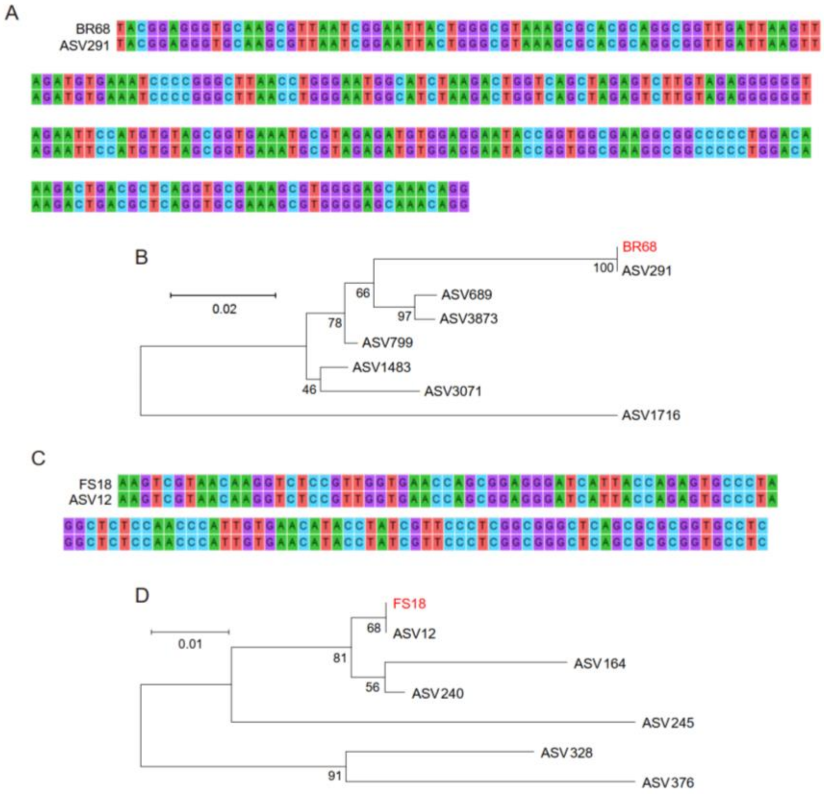

Fig. S10 (A) Sequence alignment and (B) phylogenetic tree between strain BR68 and BAC_ASV291 in bacterial communities. (C) Sequence alignment and (D) phylogenetic tree between strain FS18 and FUN_ASV12 in fungal communities.

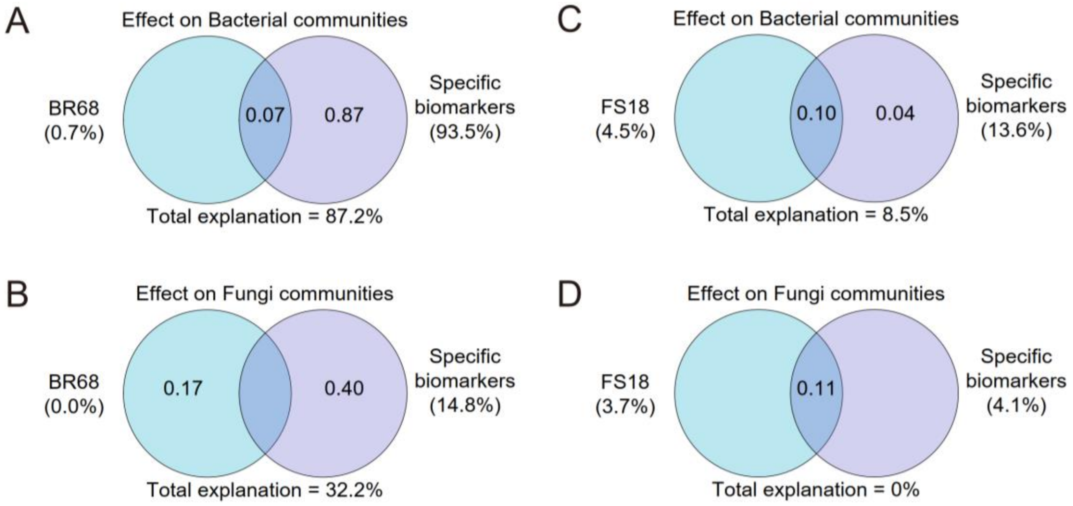

Fig. S11 In Group BR, the VPA evaluated the explanatory power of the abundance of strain RB68 and specific biomarkers to the variation of (A) bacteria and (B) fungal communities. In Group FS, the VPA evaluated the explanatory power of the abundance of strain FS18 and specific biomarkers to the variation of (C) bacteria and (D) fungal communities.

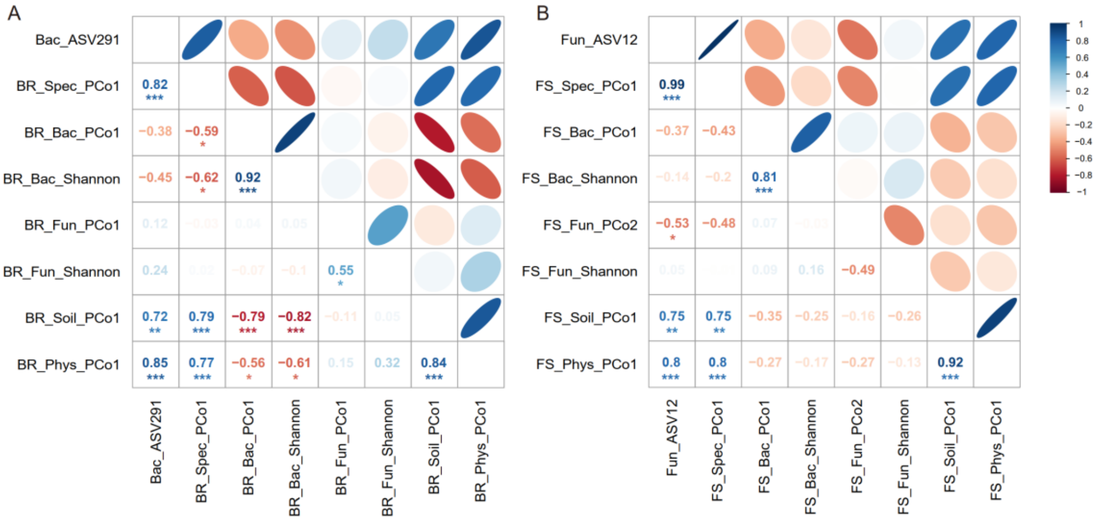

Fig. S12 (A) Spearman correlations between the abundance of strain RB68, specific biomarkers, microbial communities, soil properties and maize physiologies in Group BR. (B) Spearman correlations between the abundance of strain FS18, specific biomarkers, microbial communities, soil properties and maize physiologies in Group FS.

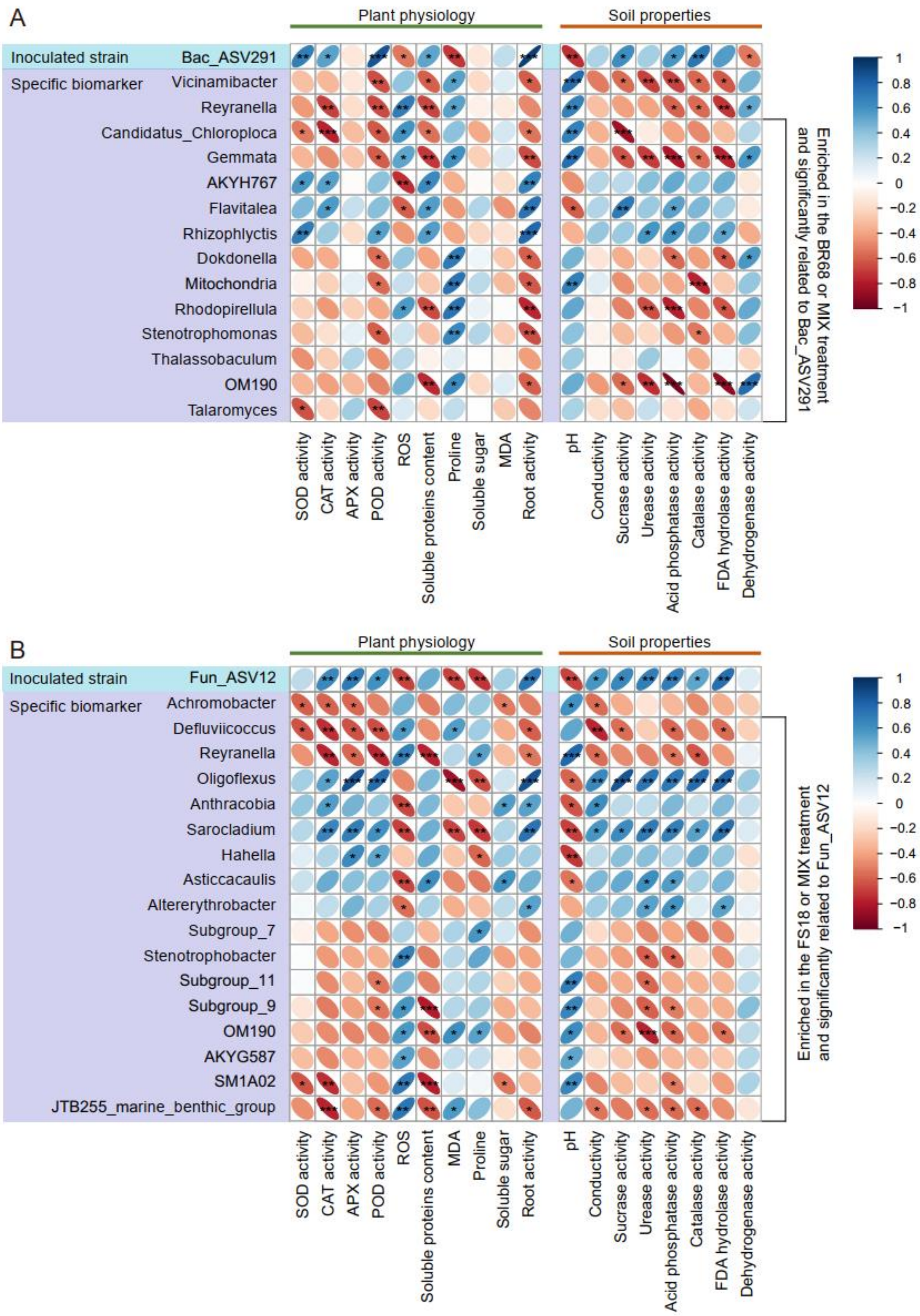

Fig. S13 (A) Spearman correlations between each of specific biomarkers and each of soil properties and maize physiologies in Group BR. (B) Spearman correlations between each of specific biomarkers and each of soil properties and maize physiologies in Group FS.
